# Synthesis, Morphology, and Crystallization Kinetics of Polyheptalactone (PHL)

**DOI:** 10.1021/acs.biomac.3c00305

**Published:** 2023-06-21

**Authors:** Maria Rosaria Caputo, Asier Olmos, Bo Li, Jorge L. Olmedo-Martínez, Anna Malafronte, Claudio De Rosa, Haritz Sardon, Rachel K. O’Reilly, Andrew P. Dove, Alejandro J. Müller

**Affiliations:** † POLYMAT and Department of Polymers and Advanced Materials: Physics, Chemistry and Technology, Faculty of Chemistry, University of the Basque Country UPV/EHU, Paseo Manuel de Lardizábal, 3, 20018 Donostia-San Sebastián, Spain; ‡ University of Birmingham, Edgbaston, Birmingham B15 2TT, United Kingdom; § Dipartimento di Scienze Chimiche, Università di Napoli Federico II, Complesso Monte S. Angelo, Via Cintia, 80126 Napoli, Italy; ∥ IKERBASQUE, Basque Foundation for Science, Plaza Euskadi 5, 48009 Bilbao, Spain

## Abstract

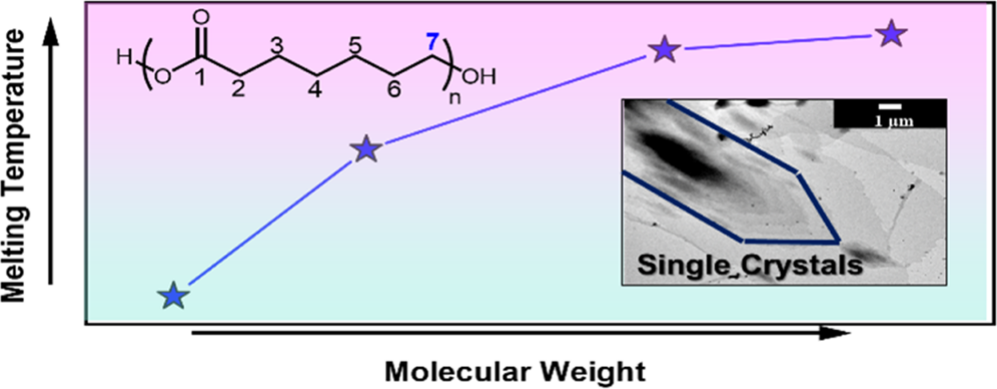

Aliphatic polyesters are widely studied due to their excellent properties and low-cost production and also because, in many cases, they are biodegradable and/or recyclable. Therefore, expanding the range of available aliphatic polyesters is highly desirable. This paper reports the synthesis, morphology, and crystallization kinetics of a scarcely studied polyester, polyheptalactone (PHL). First, we synthesized the η-heptalactone monomer by the Baeyer–Villiger oxidation of cycloheptanone before several polyheptalactones of different molecular weights (in the range between 2 and 12 kDa), and low dispersities were prepared by ring-opening polymerization (ROP). The influence of molecular weight on primary nucleation rate, spherulitic growth rate, and overall crystallization rate was studied for the first time. All of these rates increased with PHL molecular weight, and they approached a plateau for the highest molecular weight samples employed here. Single crystals of PHLs were prepared for the first time, and hexagonal-shaped flat single crystals were obtained. The study of the crystallization and morphology of PHL revealed strong similarities with PCL, making PHLs very promising materials, considering their potential biodegradable character.

## 1 Introduction

Plastic materials are widely used for their versatility, low production cost, and easy manufacturing. Furthermore, plastic materials possess a wide range of properties, so it is possible to find them in almost all production sectors, such as packaging, clothing, medicine, and electronics.^1−3^ On the other hand, the massive production and use of plastics have led to one of the main problems of the last decades: plastic waste and its disposal.^4−7^ Another negative aspect is that these plastic materials are petroleum derivatives, which increases the emission of CO_2_ into the atmosphere and affects climate change.^8^ To overcome this problem, recycling these plastic materials to obtain value-added products has been used to manage issues associated with their disposal.^9−14^ Additionally, producing and using materials with lower environmental impact is an alternative.^15,18,16,17^

Polyesters are conventionally processed in various forms, such as fibers, filaments, resins, etc., and have broad applications across packaging, textiles, automotive, medical, electronic, and construction fields. Some polyesters can be recycled through physical (mechanical) and chemical (hydrolysis, methanolysis, and glycolysis reaction) methods, and their recycled parts can also be used in the packaging and construction fields. Efficient and effective recycling of polyesters could lead to the reduction of CO_2_ emissions and, consequently, of global warming.^17,18^

A versatile aliphatic polyester that is also biodegradable is polycaprolactone (PCL), which is usually obtained by ring-opening polymerization (ROP) of ε-caprolactone or by polycondensation of hydroxycaproic acid.^19^ PCL is a biocompatible and biodegradable polymer; it is miscible with several other polymers,^20−22^ and the costs associated with its production are very low. These advantageous properties make PCL one of the most used polyesters and suitable for different fields of application, such as tissue engineering, drug delivery systems, or as an additive for polyurethanes.^23−26^

Similar alternative polymeric materials are being investigated, considering the advantages of aliphatic polyesters. Poly(η-heptalactone) (PHL), a polyester derived from η-heptalactone (lactone that contains one more carbon atom than ε-caprolactone), has been largely understudied thus far, as it is not commercially available. Given the similarity in structure to ε-caprolactone (PCL), PHL could be expected to exhibit comparable properties in processability, melting, and crystallization temperatures. Another potentially interesting aspect could be the presence of one more carbon atom than PCL, which could affect the crystallization. For example, in a recently published study,^26^ it was reported that PEB (poly-(ethylene brassylate)), a short-long aliphatic polyester (with 13 methylene groups in its repeat unit), exhibits a peculiar crystallization behavior due to a similar self-poisoning effect to that observed previously in long-chain alkanes.^26−29^ The self-poisoning effect consists in the display of a crystallization rate minima upon decreasing the isothermal crystallization temperature. In the case of long-chain alkanes, extended-chain lamellae form first at high crystallization temperature, then segments with the folded chain conformation attach, upon decreasing *T*_c_ values, to the growth front, preventing further growth until they detach, and growth can again proceed in the extended form.^26−29^ The higher ratio of methylene groups versus ester groups in PEB along the aliphatic chain leads to properties that come closer to that of polyethylene (PE), as the polar group is “diluted” along the nonpolar rest of the chain. In this sense, PEB is closer to PE, while PHL is closer to PCL.

Only a few reports have shown the ability to synthesize PHL; all have resulted in polymers with large dispersity values (2.8).^30^ The synthesis and characterization of low polydispersity (PHL) with predictable molecular weights and a study of the crystallization of these interesting materials have not been reported previously in the literature. Therefore, in this work, we report the synthesis and thermal and structural characterization of polyheptalactone (PHL). The effect of the PHL molecular weight on its thermal properties, nucleation, and crystallization kinetics is studied for the first time. Additionally, we report, also for the first time, the preparation of single crystals of this scarcely studied semicrystalline polymer. Studying crystallization, morphology, and crystallization kinetics is very important as these aspects influence properties such as thermal properties, mechanical behavior, and biodegradation potential.

## 2 Experimental Section

### 2.1 Materials

Chemicals and solvents were purchased from Sigma-Aldrich, Acros, Fluka, Fisher Chemical, Alfa Aesar, or VWR. Dry solvents were purified using the MBRAUN SPS solvent purification system. η-Heptalactone was dried over calcium hydride for 24 h before vacuum distillation. The dual-functional chain transfer agent (CTA), 4-cyano-4-(((ethylthio)carbonothioyl)thio)pentanoic acid (CEPA), was prepared according to a previously reported procedure.^36^

### 2.2 Synthesis of η-Heptalactone

As η-heptalactone is not commercially available, it was synthesized here by the Baeyer–Villiger oxidation method according to previously reported literature.^31^ Briefly, the cycloheptanone (223 mmol) and *m*-chloroperbenzoic acid (275 mmol) were mixed in CH_2_Cl_2_ (250 mL). The suspension was heated under reflux for 3 days. The reaction mixture was cooled in an ice bath, and the solids were filtered over Celite and washed with CH_2_Cl_2_ (2 × 50 mL). The filtrate was washed with 10% Na_2_S_2_O_3_ solution (2 × 200 mL), saturated Na_2_CO_3_ solution (2 × 200 mL), and saturated NaCl solution (1 × 200 mL). The organic layer was dried with MgSO_4_, filtered, and evaporated in vacuo. The resulting liquid was distilled over CaH_2_ to afford the lactone in yields of around 70%.

^1^H NMR (400 MHz, 298 K, CDCl_3_): δ = 4.28 (t, 2H, C*H*_2_O), 2.48 (t, 2H, C*H*_2_C=OO), 1.75 (m, 4H, C*H*_2_), 1.52 (m, 4H, C*H*_2_) ppm. ^13^C NMR (125 MHz, 298 K, CDCl_3_): δ = 176.4 (O*C*OCH_2_), 64.3 (OCO*C*H_2_), 31.0 (*C*H_2_COO), 30.5 (OCH_2_*C*H_2_), 28.0 (*C*H_2_CH_2_COO), 25.4 (*C*H_2_CH_2_ CH_2_COO) and 23.5 (OCH_2_CH_3_*C*H_2_) ppm.

### 2.3 Synthesis of Poly(η-heptalactone)

Four different homopolymers of polyheptalactone (PHL) with different molecular weights were prepared in this study. Briefly, in a nitrogen-filled glovebox, solutions of diphenylphosphate (10 mg, 0.04 mmol) in dry toluene (1 mL) and CEPA (9.92 mg, 0.04 mmol) in dry toluene (1 mL) were added to η-heptalactone (490 μL, 4 mmol). After stirring at room temperature for a defined time period, the solution was removed from the glovebox, precipitated three times into ice-cold methanol, and collected by centrifugation. ^1^H NMR (400 MHz, CDCl_3_) δ/ppm: 4.04–4.11 (t, C*H*_2_OH), 3.63 (m, C(CN)C*H*_2_CH_2_), 3.33 (q, SC*H*_2_CH_3_), 2.28 (t, OCOC*H*_2_), 1.61–1.35 (m, OCOCH_2_(C*H*_2_)_3_CH_2_OH).

### 2.4 ^1^H Nuclear Magnetic Resonance (NMR)

Unless otherwise stated, ^1^H nuclear magnetic resonance (NMR) spectra were recorded at 400 MHz on a Bruker DPX-400 spectrometer in CDCl_3_. Chemical shifts are reported as δ in parts per million (ppm) downfield from the internal standard trimethylsilane.

### 2.5 Size Exclusion Chromatography (SEC)

Size exclusion chromatography (SEC) was performed on an Agilent 390-MDS on PLgel Mixed-D type columns in series with refractive index (RI) detection. Weights were calculated using a calibration curve determined from poly(styrene) standards with chloroform (0.5% NEt3) as eluent flowing at 1.0 mL.min^–1^ and sample concentration of 3 mg mL^–1^.

### 2.6 Thermogravimetric Analysis

To determine the temperature at which the samples thermally degrade in air, a PerkinElmer thermogravimetric analyzer was used. To perform this experiment, 10 mg of each sample were placed in a platinum crucible and were heated from 30 to 600 °C at 10 °C/min.

### 2.7 Differential Scanning Calorimetry (DSC)

A PerkinElmer Pyris 8000 DSC with an Intracooler 2P was employed to characterize thermal properties. The instrument was previously calibrated with indium and tin standards. Samples of 5 mg for each PHL were used, and the experiments were carried out under ultrapure nitrogen flow by placing the materials in sealed aluminum pans. Non-isothermal and isothermal experiments were performed.

During the non-isothermal experiments, the samples were heated up to 90 °C at 20 °C/min and left at this temperature for 3 min to erase the thermal history; then, they were cooled at 20 °C/min down to 25 °C and held for 1 min at this temperature. Afterward, they were heated to 90 °C/min at 20 °C/min.

During the second heating scan, it was possible to calculate the degree of crystallinity of each PHL sample as follows
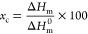
where Δ*H*_m_ (J/g) is the melting enthalpy of the samples and Δ*H*_m_^0^ is the enthalpy of melting for a fully crystalline sample (195 J/g) calculated according to the group contribution method.^32^

To investigate the overall crystallization kinetics, isothermal experiments were carried out. In the first part of the experiment, the minimum isothermal crystallization temperature *T*_c,min_ was determined by the trial and error method proposed by Lorenzo et al.^33^ Samples of PHL were cooled from the melt to *T*_c_ values at 60 °C/min and immediately reheated at 20 °C/min up to 90 °C. This protocol was repeated cyclically at decreasing *T*_c_ until no melting phenomenon was found in the reheating scan. In this way, it was possible to determine a *T*_c_ range. The isothermal crystallization experiments were performed following the procedure suggested by Lorenzo et al.:^33^ (I) heating from room temperature to 30 °C above the melting point at 20 °C/min, i.e., 90 °C; (II) holding the sample for 3 min at 90 °C to erase thermal history; (III) quenching the sample to a predetermined crystallization temperature (*T*_c_) at 60 °C/min; (IV) isothermal crystallization until maximum saturation (in our case between 15 and 60 min for each *T*_c_); and (V) heating from *T*_c_ to 90 °C at 20 °C/min to record the melting behavior after the isothermal crystallization. This final melting run provided the values of apparent melting points employed to perform the Hoffman–Weeks extrapolation to calculate the equilibrium melting temperature of each material. These experiments were performed on as-prepared samples, which were in powder form.

### 2.8 X-ray Powder Diffraction of the As-Prepared and Melt-Crystallized Samples

X-ray powder diffraction profiles of the as-prepared (precipitated from the polymerization solution) and melt-crystallized samples of PHL were obtained at room temperature with Ni-filtered Cu Kα radiation (λ = 1.5418 Å) by using an automatic Philips diffractometer operating in the reflection geometry. Melt-crystallized compression-molded samples were prepared by heating the as-synthesized samples at 30–40 °C above the melting temperature between the plates of a press and then cooling to room temperature by circulating cold water inside the press plates (estimated cooling rate ≈ 20 °C/min).

### 2.9 Preparation and Characterization of Single Crystals of PHL

A 0.012 wt % solution of the sample PHL 66 in 1-hexanol was prepared by dissolving the polymer (0.3 mg) into 3 mL of solvent. The solution was placed at 85 °C and maintained at this temperature for 1 h to completely dissolve the polymer. The solution was then slowly cooled to 50 °C (estimated cooling rate ≈ 1.2 °C min^–1^) and kept at this temperature for 21 h to allow crystallization. Afterward, the solution was slowly cooled to room temperature (estimated cooling rate lower than 1 °C min^–1^). Drops of crystal suspension were deposited on carbon-coated grids and allowed to dry before transmission electron microscopy (TEM) analysis. Bright-field TEM images of crystals were acquired using an FEI TECNAI G2 200 kV S-TWIN microscope (electron source with LaB_6_ emitter) operating at 120 kV.

### 2.10 Polarized Light Optical Microscope (PLOM) Analysis

A polarized light optical microscope, Olympus BX51 (Olympus, Tokyo, Japan), equipped with an Olympus SC50 digital camera and with a Linkam-15 TP-91 hot stage (Linkam, Tadworth, U.K.) (coupled to a liquid nitrogen cooling system) was used to observe the morphology of the samples, after crystallization from the melt. Films with approximately 50 μm thickness were prepared by melting the samples between two glass slides.

The samples were heated to 90 °C, kept at this temperature for 3 min to erase their thermal history, and then cooled from the melt at 20 °C/min to 25 °C; using the same equipment, the isothermal spherulitic growth rate was measured. The samples were heated between two glass slides to 90 °C and kept at this temperature for 3 min to erase the thermal history. The samples were then cooled at 50 °C/min to a temperature where the spherulites began appearing. The spherulite growth was followed isothermally as a function of time by recording micrographs. The procedure was repeated for different temperatures, and for each temperature, the radius of the spherulites was measured and plotted as a function of time. From these data, the growth rate (*G*) of the spherulites was determined, and the experimental values of *G* versus *T*_c_ were fitted using the Lauritzen–Hoffman equation.^34^

### 2.11 Transmission Electron Microscopy (TEM)

TEM analysis was conducted to observe the morphology at the lamellar level. A RuO_4_ solution was used for staining PHL films of roughly 1 mm thickness. Thin strips of samples were put into this solution for 16 h. Afterward, ultra-thin sections were cut at −90 °C with a diamond knife with a Leica EMFC6 ultra-cryo-microtome device. The ultra-thin, 90 nm thick sections were mounted on 200 mesh copper grids. Finally, they were examined using a TECNAI G2 20 TWIN TEM equipped with a LaB_6_ filament operating at an accelerating voltage of 120 kV.

## 3 Results and Discussion

As η-heptalactone (HL) is not commercially available, it was first synthesized and characterized. The first aim was to obtain poly(η-heptalactone) (PHL) with predictable molecular weight and low dispersity. Therefore, ROP kinetics of the HL polymerization was studied as catalyzed by an organocatalyst, namely, diphenylphosphate. A range of different DPs of PHL homopolymers were targeted for further investigation of crystallization kinetics.

### 3.1 Synthesis of Poly(η-heptalactone)

**Figure 1:**
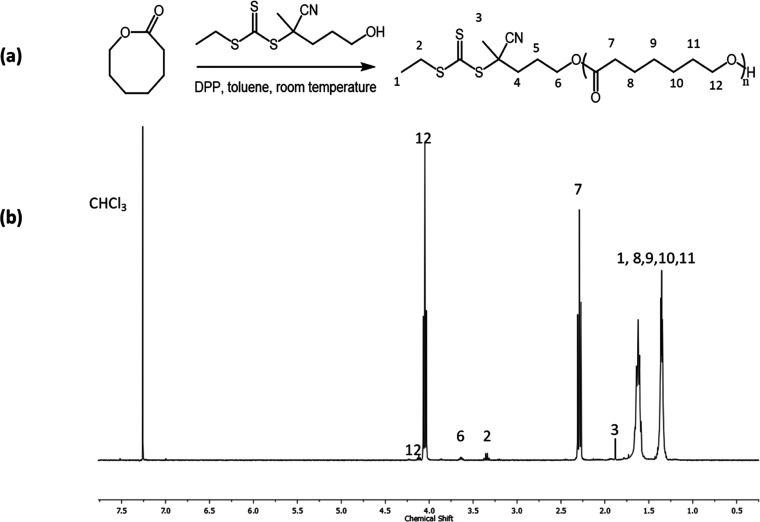
(a) Synthesis of the PHL polymers by ROP of η-heptalactone catalyzed by DPP and (b) ^1^H NMR spectrum of PHL 35 (CDCl_3_, 400 MHz).

η-Heptalactone was synthesized by Baeyer–Villiger oxidation following a literature procedure. The pure product was confirmed with ^1^H NMR spectroscopy and was consistent with previous literature data (Figure S1).^31^ After the successful preparation of the monomer, the homopolymerization of η-heptalactone was carried out. Diphenylphosphate (DPP) was selected as the catalyst as a result of the superior ability of this catalyst to provide polylactones with narrow dispersities and end-group fidelity.^35^ The polymerization was initiated by a dual-head initiator and chain transfer agent to be consistent with our previously reported PCL work^36^ (Figure 1a).

The ROP of η-heptalactone was attempted at a monomer concentration of 4 M in toluene as solvent at room temperature in an N_2_-filled glovebox, with initiator (DP_tot_ = 100) and 1 mol % DPP catalyst. ^1^H NMR spectroscopic analysis was employed to monitor the reactions and characterize the resulting polymers (Figure 1b). The disappearance of the characteristic signals of the methylene protons of the HL monomer at δ = 4.28 ppm and the concomitant appearance of these methylene protons in the polymer chain at δ = 4.05 ppm permit us to calculate the conversion. The polymerization reached 70% conversion after 4 h while still exhibiting first-order kinetics (Figure S2). Using these conditions, different DPs of PHL were targeted for further crystallization study. Different conversion degrees were obtained depending on the reaction time, and therefore four different samples of PHL with different molecular masses were synthesized (Table 1).

**Table 1:** Polymerization Time, Degree of Conversion, Average Molecular Masses, and Polydispersity of the Synthesized Samples of PHL

time (h)	sample	conversion (%)	*M*_n_^a^_(GPC)_ (kDa)	*M*_w_^a^_(GPC)_ (kDa)	*Đ*_M_	*M*_n [^1^H (NMR)]_^b^ (kDa)
1	PHL 15	15	6.2	7.0	1.11	2.2
2	PHL 35	35	9.9	11.4	1.11	4.7
4	PHL 66	70	16.6	19.6	1.15	9.2
5	PHL 90	90	20.8	22.7	1.17	11.8

a Measured by SEC.

b Measured by ^1^H NMR spectroscopy.

The average molecular weight was measured by ^1^H NMR spectroscopy and SEC. The polymer molecular weights were determined by end-group analysis by ^1^H NMR spectroscopy, comparing the ratio between the polymer CH_2_OC=O resonances of (δ = 4.05) and the chain transfer reagent SCH_2_CCN(CH_3_) resonance (δ = 3.69). Size exclusion chromatography (SEC) analysis is reported in the SI and reveals low dispersity (*Đ*_M_ < 1.2) and good overlap of the refractive index (RI) and ultraviolet (UV) (λ = 309 nm, corresponding to the π–π* electronic transition of the thiocarbonyl moiety) peak in the SEC traces, which signifies the retention of the thiocarbonyl group in the initiator (Figure S3).

### 3.2 X-ray Powder Diffraction

**Figure 2:**
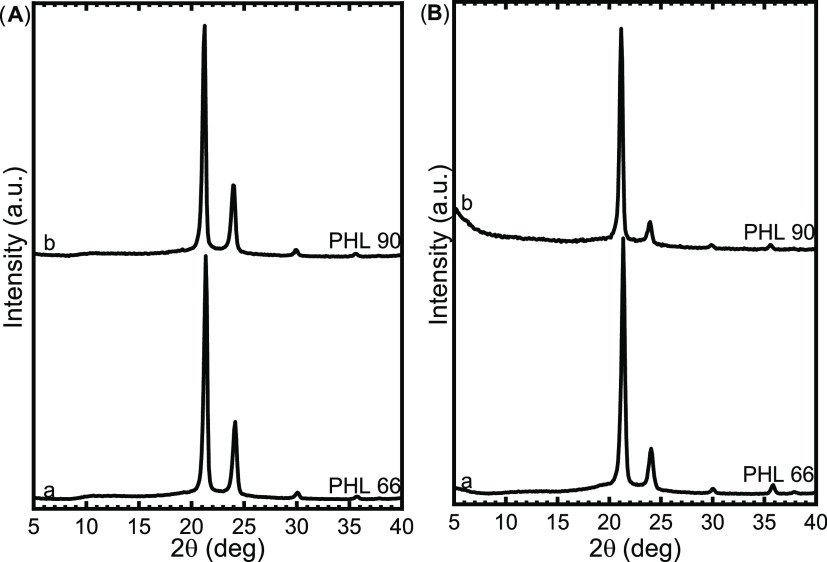
X-ray powder diffraction profiles of the as-prepared (A) and melt-crystallized (B) specimens of samples PHL 66 (a) and PHL 90 (b).

The X-ray powder diffraction profiles of the as-prepared and melt-crystallized samples of PHL of higher molecular mass (samples PHL 66 and PHL 90), acquired in the 2θ region between 5 and 40°, are shown in Figure 2a,b, respectively.

The diffraction profiles of both as-prepared and melt-crystallized samples (Figure 2a,b) are characterized by two strong and sharp reflections at 2θ ≈ 21 and 24° and other minor diffraction peaks of lower intensity in the 2θ region between 25 and 40°. Similar diffraction profiles have been obtained for the different samples in Table 1. The X-ray diffraction profiles of PHL (Figure 2) appear similar to that of polyethylene (PE)^37^ and other linear aliphatic polyesters, ―(―O―(CH_2_)_*m*_―CO―)_*n*_―, such as poly(11-undecalactone) (PUDL, *m* = 10),^38^^38^ poly(δ-valerolactone) (PVL, *m* = 4),^39^ poly(β-propiolactone) (PPL, *m* = 2),^40^ poly(16-hexadecalactone) (PHDL, *m* = 15),^41^ poly(12-dodecalactone) (PDDL, *m* = 11),^42^ poly(15-pentadecalactone) (PPDL, *m* = 14),^43^ and poly(*ε*-caprolactone) (PCL, *m* = 5).^44,45^

### 3.3 TGA Results

**Figure 3:**
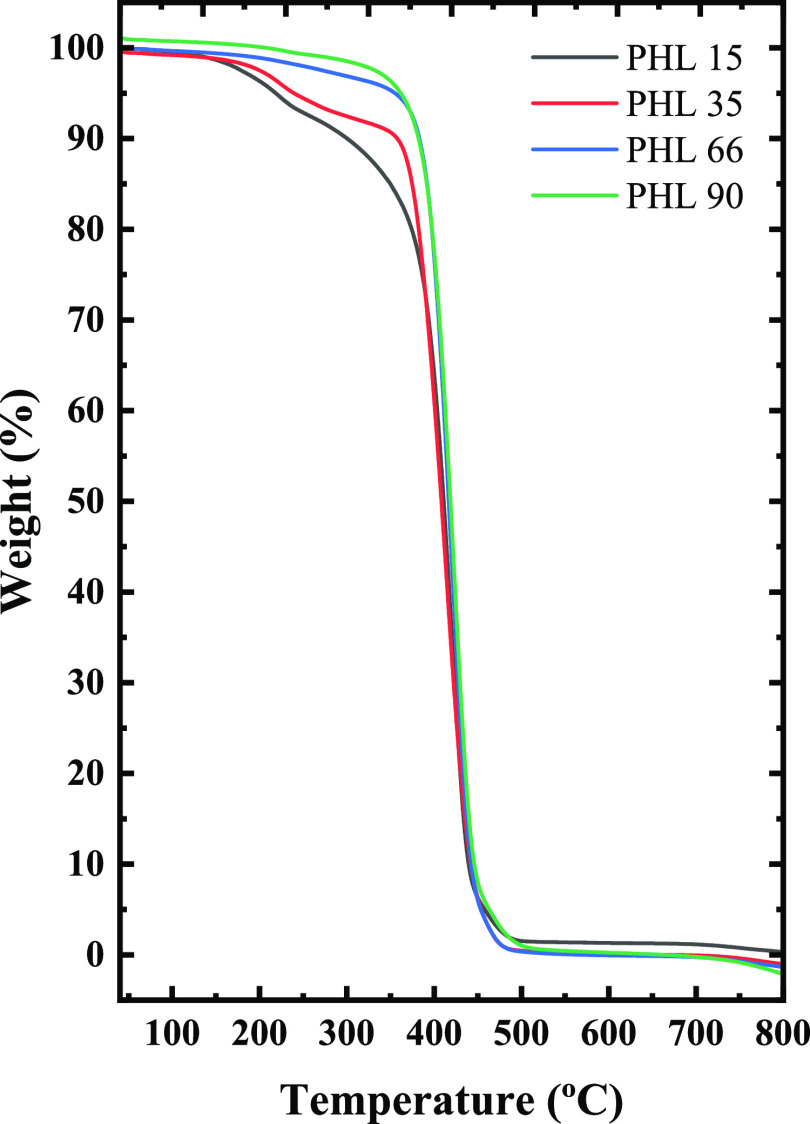
Weight loss (%) as a function of the temperature (°C) for PHL samples.

Figure 3 shows the weight loss profiles of the four PHL samples revealed by TGA. In the case of the two samples with the lowest molecular weight (PHL 15 and PHL 35), the TGA curves are characterized by a two-step weight loss at about 210 and 380 °C. This phenomenon disappears in the two samples with the highest molecular weight (PHL 66 and PHL 90), with only one weight loss step at about 400 °C. This particular behavior has already been observed previously in the case of PCL samples with different molecular weights,^46^ in which the reason for the first step of weight loss for low-molecular-weight samples was initially attributed to the development of ε-caprolactone in the thermal decomposition process, and FT-IR spectroscopic analyses subsequently confirmed this. Since PCL is chemically similar to PHL, it is possible that the behavior detected by the TGA could be attributed to the same cause, i.e., the production of η-heptalactone during the thermal degradation process.

### 3.4 Non-Isothermal DSC

**Figure 4:**
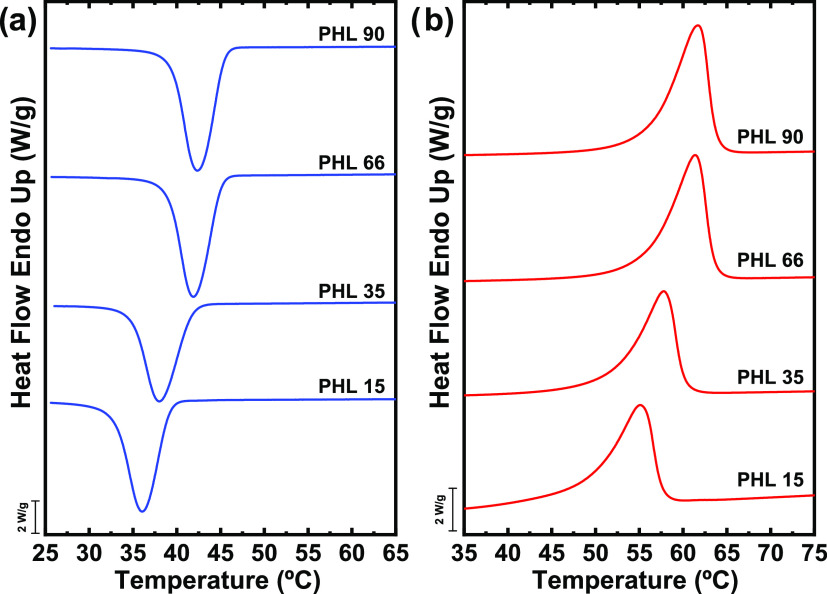
(a) DSC thermograms (cooling scans) at 20 °C/min and (b) subsequent DSC thermograms (heating scans) at 20 °C/min for PHL samples.

**Figure 5:**
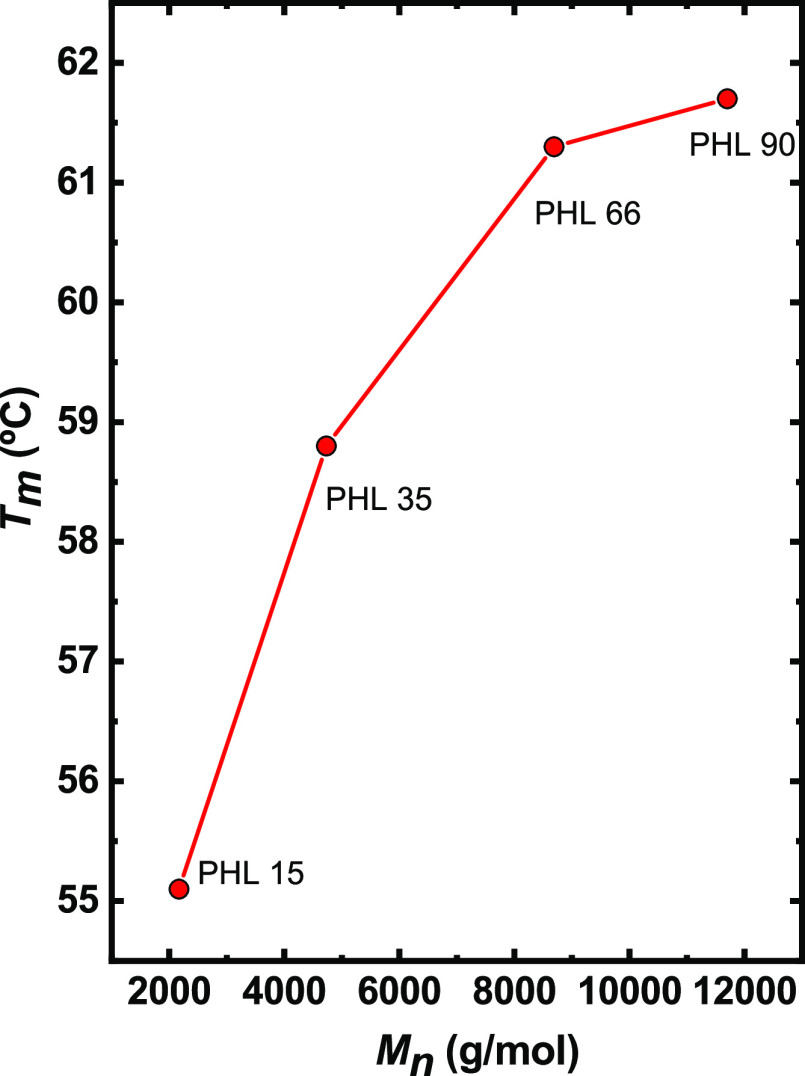
Melting temperatures as a function of molecular weight of PHL samples; the solid red line is a line to guide the eye.

Figure 4 shows DSC results obtained during cooling from the melt and subsequent heating scans of the different molecular weight PHL samples. It is observed that the crystallization temperature increases as a function of molecular weight, from 36 °C for the lowest molecular weight to 42.5 °C for the highest molecular weight. The melting temperature has a similar behavior, increasing as a function of molecular weight from 55 to 61.5 °C. Figure 5 shows the change in *T*_m_ as a function of molecular weight. As expected, the melting temperature increases with molecular weight but does not reach saturation in the range of molecular weights explored here. The same trend is observed for *T*_c_, as reported in Figure S4.

The values of the melting and crystallization temperatures with the relative enthalpies are shown in Table S1 of the Supporting Information. The same table also shows the glass-transition temperature (*T*_g_), and degree of crystallinity (*x*_c_) values obtained in the second heating scan. Figure S5 reports an enlargement of the second heating scans to appreciate the glass transition better.

### 3.5 Morphology and Spherulitic Growth

**Figure 6:**
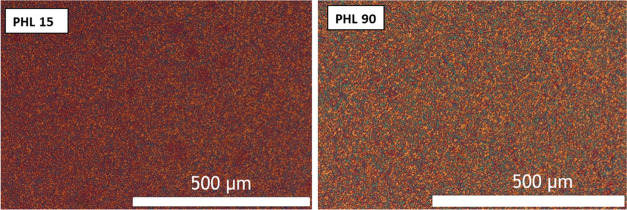
PLOM micrographs for PHL samples. Micrographs were taken at 25 °C after melting for 1 min at 90 °C and cooling at 20 °C/min.

The samples were analyzed by PLOM to study their superstructural morphology. Figure 6 shows the PLOM micrographs using the same magnification scale bar (500 μm) collected at 25 °C after non-isothermal crystallization from the melt at 20 °C/min. The micrographs of the samples with the lowest and highest molecular weight (PHL 15 and PHL 90) are reported since the morphology does not show significant differences for the other samples. Samples crystallize, forming microspherulites, regardless of their molecular weight. The results imply that the samples possess a very high nucleation density, probably coming from catalytic debris, which can act as very active nucleating heterogeneities.

**Figure 7:**
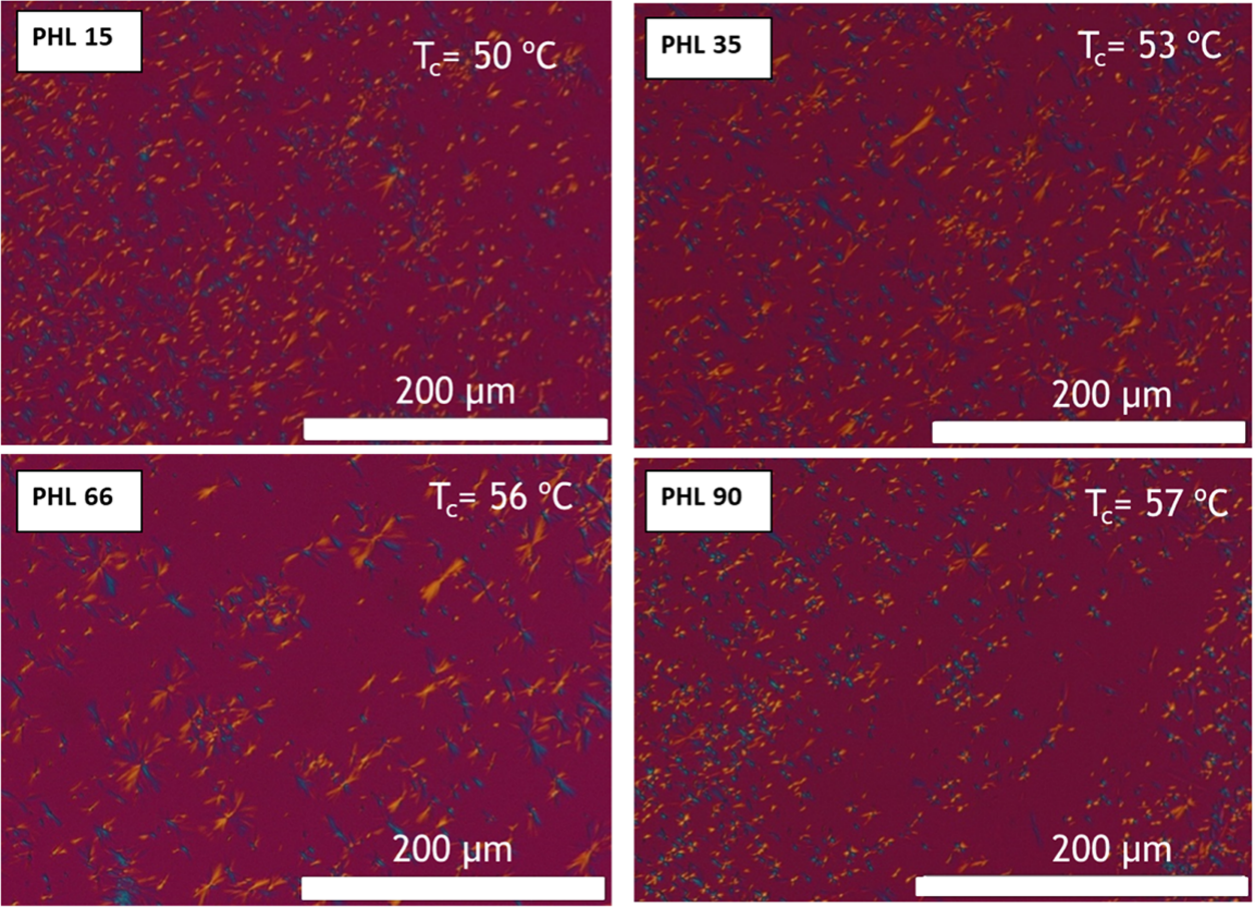
PLOM micrographs taken at the indicated *T*_c_ values and at the same supercooling (Δ*T* = 5 °C). The rate of cooling from the melt was 20 °C/min.

When the samples are isothermally crystallized, the morphology can be better appreciated, as heterogeneous nuclei activation is known to decrease when the isothermal crystallization temperature increases. Figure 7 shows the PLOM micrographs acquired during isothermal crystallization experiments at the indicated *T*_c_ and with the same supercooling value (the equilibrium melting temperature needed to calculate the supercooling was also obtained in this work; see below). The samples were cooled from the melt (at 50 °C/min) to a chosen crystallization temperature in the range of 45–60 °C. Although the crystals appear larger, they do not have the classic circular shape typical of spherulites: they resemble more axialites or lamellar aggregates with two-dimensional symmetry. As their average sizes are close to one another, the nucleation is probably instantaneous, as was later confirmed by the overall crystallization kinetics measurements (see below).

Given the shape of the crystals, to measure the growth rate, *G* (μm/s), the length variation from one extreme to the other was measured as a function of time for different crystallization temperatures (the plots were always linear).

The typical growth rate (*G*) trend as a function of temperature is represented by a bell-shaped curve, given by the competition of two opposite phenomena.^47,48^ On the right-hand side of the plot, the growth rate increases as *T*_c_ decreases because the secondary nucleation increases with the supercooling until a maximum (i.e., *G* is controlled by secondary nucleation in this high-temperature range). This maximum corresponds to the point at which the viscosity of the melt is so high that the growth of the crystals is dominated by the slow diffusion of the chains to the crystallization front. Subsequently, the growth rate decreases and becomes zero at *T*_g_.

In the case of the PHL samples involved in this study, it was possible to measure growth rates only on the right side of the *G* versus *T*_c_ curve at high crystallization temperatures. Any attempt to analyze growth measurements at lower temperatures failed, as the material crystallized during the fast cooling to *T*_c_.

**Figure 8:**
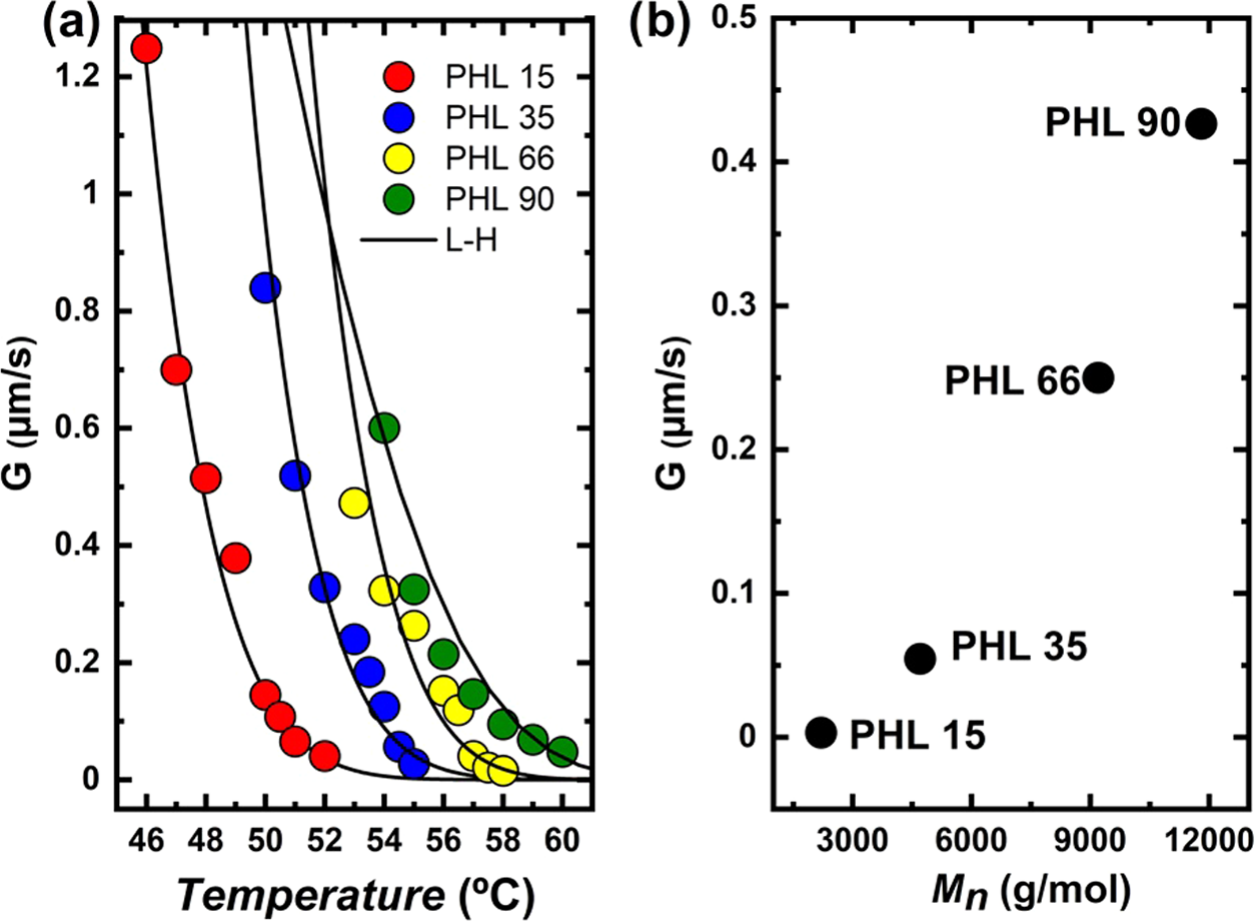
Spherulitic growth rate (*G*) as a function of: (a) crystallization temperature and (b) molecular weight at *T*_c_ = 54.5 °C for samples PHL 15, PHL 35, PHL 66, and PHL 90. The solid lines in the left graph are fits to the Lauritzen and Hoffman equation.

Figure 8a shows the results of the spherulitic growth rate as a function of *T*_c_ for the four samples involved in this work, determined by PLOM experiments. The trend is similar for all samples and is the typical trend where secondary nucleation dominates the superstructural growth. It is possible to notice that the molecular weight of each sample influences the growth rate values. Indeed, the growth rate is faster as the molecular weight increases, and the spherulites of the higher molecular weight samples grow faster at the same crystallization temperature in the molecular weight range studied in this work. This behavior is evident from the graph shown in Figure 8b, in which the growth rate is represented as a function of *M*_n_ at the same crystallization temperature. This result is consistent with the control of secondary nucleation. It has already been reported in the case of other low-molecular-weight polyesters, such as PCL,^49−51^ PEO,^52^ and PDEO.^53^ If higher molecular weight samples could be obtained, eventually, diffusion factors will take over, and the growth rate would eventually decrease as the molecular weight increases.

The solid lines reported in Figure 8a are fits to the Lauritzen and Hoffman theory.

The Lauritzen–Hoffman equation is given by^54^
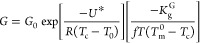
where *G*_0_ is a constant that includes all terms that do not depend on temperature, U* is the transport activation energy for the polymer chains diffusion (in this study, a value of 1500 cal/mol was employed), *R* is the universal gas constant, *T*_c_ is the crystallization temperature, *T*_0_ is the temperature at which the movement of the chains is frozen, and it is 30 °C degrees lower than the *T*_g_, *T*_m_^0^ is the equilibrium melting temperature, and *f* is a temperature correction factor, given by the expression 2*T*_c_/(*T*_m_^0^ + *T*_c_). *K*_g_^G^ is a constant proportional to the energy barrier for the spherulitic growth or secondary nucleation
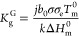
where *j* assumes the value of 2 for the Regime II crystallization, a regime where the secondary nucleation and the spread of the nucleus on the growth front are equivalent,^55^*b*_0_ is the chain’s width, σ is the lateral surface free energy, σ_e_ is the fold surface energy, *k* is the Boltzmann constant and, finally, Δ*H*_m_^0^ is the equilibrium latent heat of fusion. When  is plotted versus , it is possible to obtain a straight line in which *K*_g_^G^ is the slope, and *G*_0_ is the intercept. From the *K*_g_^G^ value, the σσ_e_ value can be obtained, and, using the expression , where *a*_0_*b*_0_ is the chain cross-sectional area, it is possible to calculate the values of σ and σ_e_. Moreover, it is also possible to calculate the work that the macromolecule does to fold as *q* = 2*a*_0_*b*_0_σ_e_.^56^

The detailed analysis of the parameters can be found in Table S2, where differences in the *K*_g_^G^, σ_e_, and *q* values are noted: these parameters decrease with increasing molecular weight. This indicates that the energy barrier for the PHL spherulites to grow decreases as *M*_n_ increases, in the molecular weight range explored in this work. A similar behavior has already been observed previously for PCL samples in a low to medium molecular weight range.^49,51^

### 3.6 Overall Crystallization Kinetics Obtained by DSC

The overall crystallization process is the transformation from the melt to the semicrystalline state, including both primary nucleation and superstructural growth. DSC is a convenient and precise technique to determine the overall isothermal crystallization kinetics. Representative DSC isotherms are reported in Figures S6a,b and S7a–d. The experimental data can be fitted with the Avrami and the Lauritzen and Hoffman theory.^57,58^

**Figure 9:**
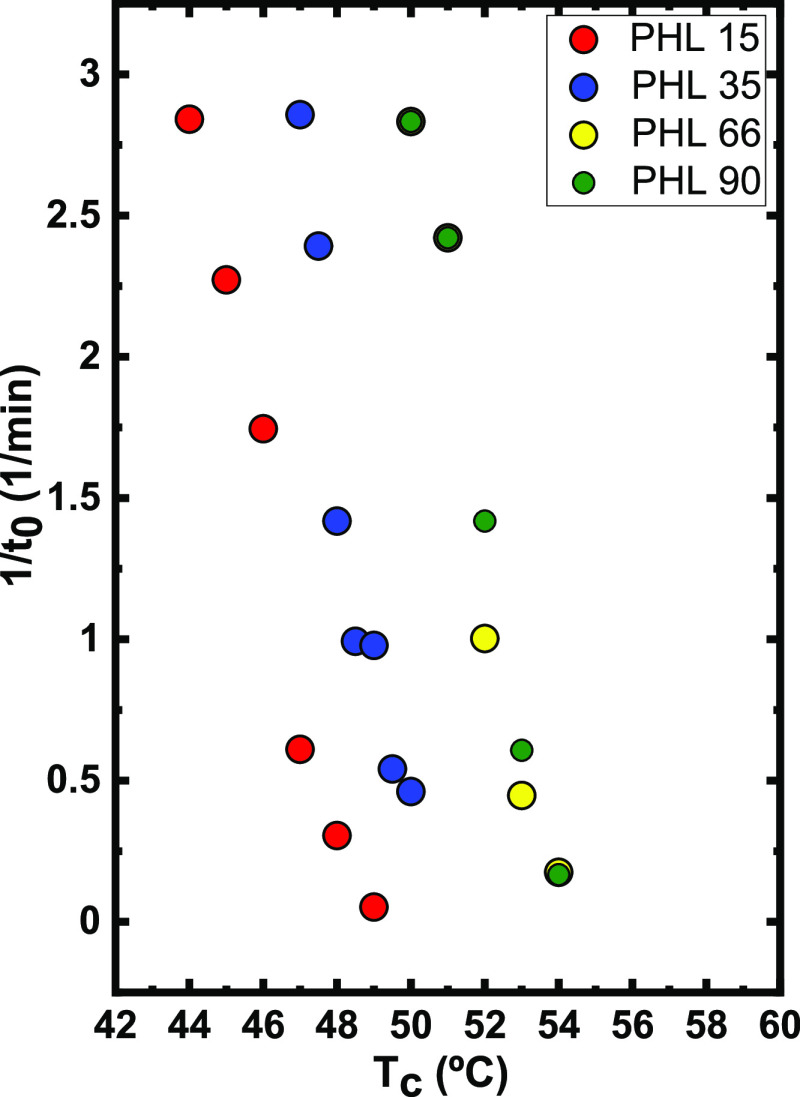
The inverse of induction time (1/*t*_0_) as a function of *T*_c_.

The inverse of the induction time (*t*_0_) is reported as a function of *T*_c_ in Figure 9. The induction time is the time that elapses before the DSC detects any crystallization process. Therefore, the inverse of the induction or incubation time is proportional to the primary nucleation rate (before crystal growth starts from the created primary nuclei). The primary nucleation rate increases (if compared at a constant temperature) as the PHL molecular weight increases. Still, the difference between PHL 66 and PHL 90 is very small, indicating a saturation effect, which was not observed for the spherulitic growth rates.

**Figure 10:**
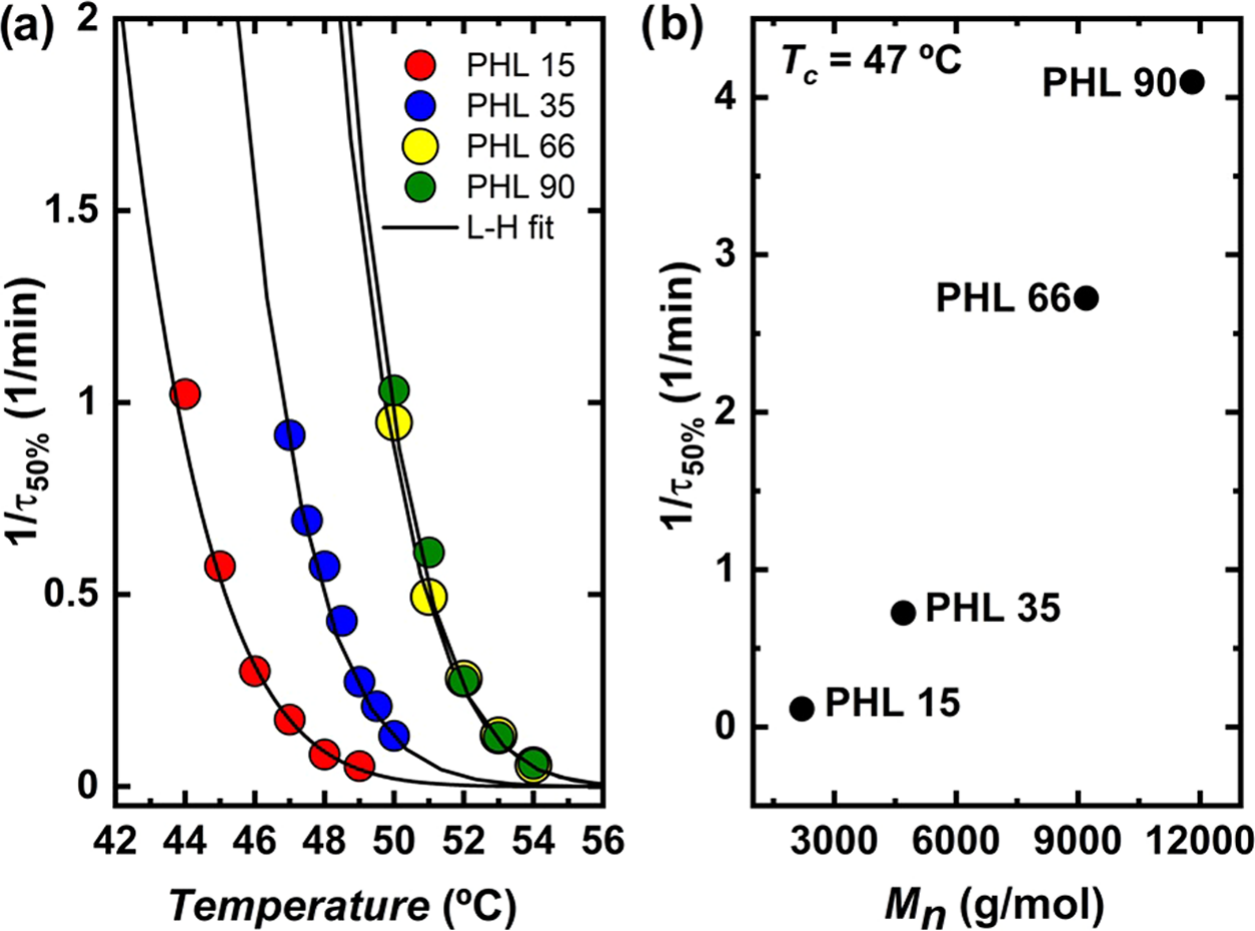
Overall crystallization rate (1/τ_50%_) as a function of (a) crystallization temperature and (b) molecular weight at *T*_c_ = 47 °C, for PHL 15, PHL 35, PHL 66, and PHL 90. The solid lines in the left graph are fits to the Lauritzen and Hoffman equation.

The inverse of the half crystallization rate (1/τ_50%_) is plotted in Figure 10 as a function of *T*_c_ (a) and molecular weight at a constant *T*_c_ (b). This value is the inverse of the time polymers need to achieve 50% of their relative crystallinity during an isothermal process. It represents an experimental value of the overall crystallization rate. The trend is similar to that seen for the inverse of the induction time, i.e., samples with higher molecular weight crystallize faster and at lower supercooling than samples with lower molecular weight. However, the difference between PHL 66 and PHL 90 is minimal in the temperature range where the experimental data was gathered. Figure 10b plots 1/τ_50%_ values as a function of the molecular weight at the same value as the crystallization temperature.

It should be noted that for the PHL 60 and PHL 90 cases, the values were extrapolated with the Lauritzen and Hoffman fits, as no experimental data for these samples could be measured at *T*_c_ = 47 °C. In any case, we have obtained a similar trend for primary nucleation rate, spherulitic growth, and overall crystallization rate; as in all cases, these rates increase with PHL molecular weight, but they are close to starting to saturate at the highest molecular weight values employed here. A similar behavior has already been observed in the case of PCL,^49,50^ PLLA,^59^ and PHB^60^ in the low to medium molecular weight range.

The experimental data of the overall DSC crystallization rate was fitted by the Avrami equation,^61−63^ which can be expressed as
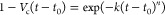
In this equation, *V*_c_ represents the relative volumetric transformed fraction, *t* is the experimental time, *t*_0_ is the induction time, *k* is the overall crystallization rate constant, and *n* is the Avrami index, related to the nucleation rate and the growth dimensionality of the crystals; the value of *n* can fluctuate between 2 and 4 in the case of bulk polymers. Avrami index values close to 2 are correlated with instantaneously nucleated axialites. If *n* = 3, two possibilities exist, either sporadically nucleated axialites or instantaneously nucleated spherulites, and finally, *n* = 4 indicates the generation of sporadically nucleated spherulites. If the nucleation is between instantaneous and sporadic, fractional values of the Avrami index can be obtained.^34^

**Figure 11:**
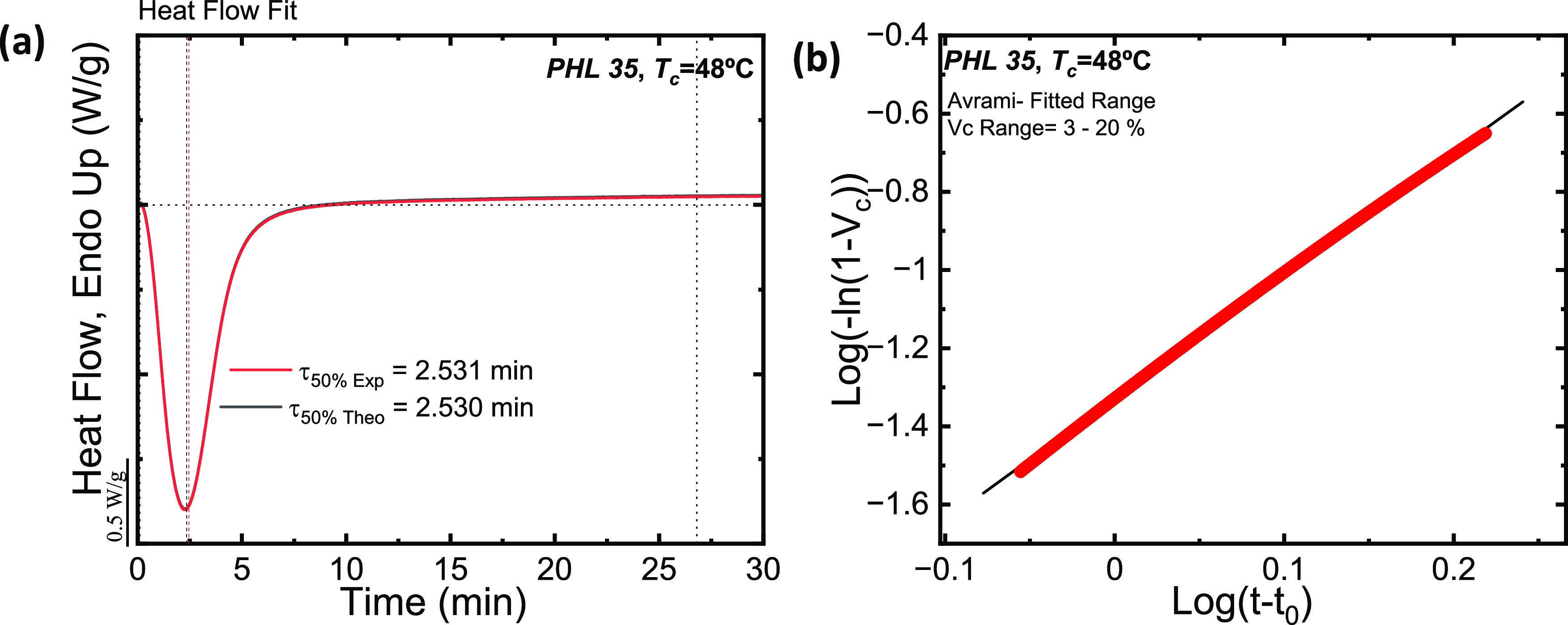
The Avrami fit equation using the free Origin plug-in developed by Pérez-Camargo et al.^34^ for PHL 35 at *T*_c_ = 48 °C.

Figure 11a shows an example of the PHL 35 sample, where the experimental data of the isothermal DSC is plotted together with its Avrami fit, and Figure 11b shows the typical Avrami plot in the conversion range used to perform the fit. The agreement between the experimental data and the Avrami fit is excellent, as in this case, the Arami equation describes not only the primary crystallization range (which is typical) but also the secondary crystallization range, after the superstructural entities (i.e., axialites most likely in this case) have impinged on one another, at experimental times beyond the peak value.

Tables S4–S7 in the SI list the fitting parameters for all of the samples employed here; it is possible to notice that the fittings obtained are always excellent with correlation coefficients larger than 0.999. The experimental values of τ_50%_ are also in excellent agreement, as expected by the quality of the fit, with those predicted by the Avrami theory. Tables S4–S7 also report the Avrami index, and it is noted that values between 2.2 and 3 were obtained. These values can be interpreted as representing axialites whose nucleation varies from close to instantaneous (*n* = 2) to sporadic (*n* = 3). In the cases where *n* = 3, the values could also represent spherulites instantaneously nucleated; however, according to the PLOM observations presented in Figure 8, they were not observed.

Moreover, in this work, during the isothermal crystallization process, the equilibrium melting temperature of the four PHL samples was calculated, and for this purpose, the Hoffman–Weeks extrapolation was used. Figure S7 shows the extrapolations obtained. No monotonic change with the molecular weight was obtained, and the equilibrium melting temperatures are in the range of 71–75 °C, which considering the error of the extrapolations performed, could be regarded as similar to one another. The average *T*_m_^0^ value obtained is close to 73 °C, similar to some of the values reported for PCL (i.e., 78 °C).^64^

### 3.7 Transmission Electron Microscopy (TEM)

**Figure 12:**
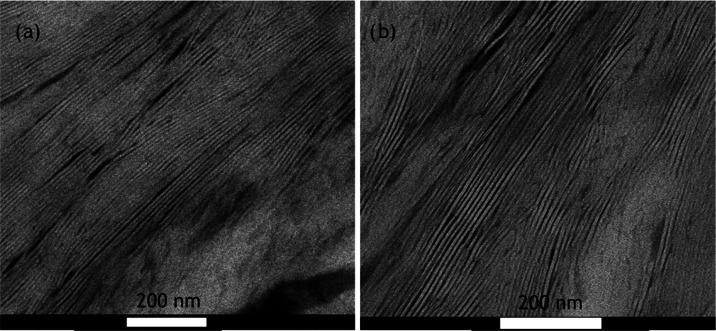
TEM micrographs taken at room temperature. Before cryo-cutting at −90 °C, the samples were crystallized by cooling from the melt at 20 °C/min.

TEM analysis was performed on the PHL 15 and PHL 35 samples; the corresponding results are shown in Figure 12a,b. In the present study, the TEM analysis was conducted after staining, so the dark areas are the amorphous areas, and the brighter areas are the crystalline areas. The presence of straight and long lamellae (white lines in Figure 12a,b) is observed, and their thickness was measured manually using ImageJ software. Figure S8 shows the thickness distribution histograms. For both samples, the lamellar thickness is very similar, with an average thickness of 8 nm, a value comparable to that obtained for PCL.^65^

**Figure 13:**
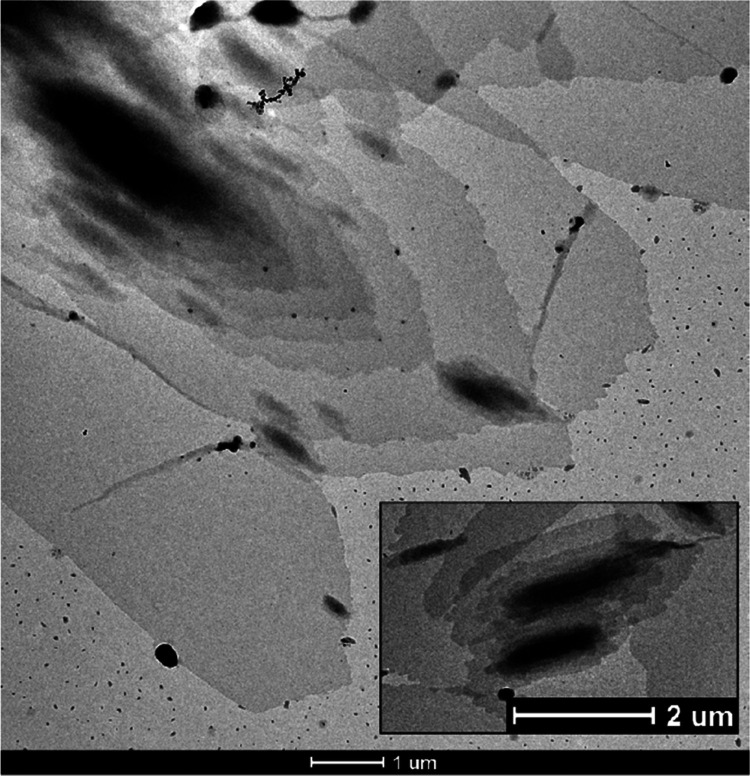
Bright-field TEM image of PHL 66 single crystals.

Single crystals of the sample PHL 66 were prepared from a dilute solution in 1-hexanol according to the procedure described in the Materials and Method section. A bright-field TEM image of the obtained crystals is reported in Figure 13.

Hexagonal-shaped flat single crystals were observed. Most crystals are multilayered. This single crystal morphology has been reported for other aliphatic polyesters, such as PUDL^38^ and PCL.^49,66,67^ Single crystals with various thicknesses were observed (Figure 13). Some crystals are transparent to the electron beam, whereas others are completely opaque. Preliminary experiments have shown that single-layered crystals, and each monolamellar part at the edge of all multilayered crystals, yield well-resolved electron diffraction (ED) patterns. The interpretation of the ED patterns and the full resolution of the crystal structure of PHL is in progress.

## 4 Conclusions

This study reports the synthesis and comprehensive crystallization study of a new polyester with seven carbon atoms in its repeating unit, i.e., polyheptalactone, PHL. The WAXS diffraction pattern of this material was collected and found to be very similar to PE and linear aliphatic polyesters. The effect of molecular weight was studied in both non-isothermal and isothermal experiments. It was found that high molecular weights correspond to materials with higher melting temperatures and faster nucleation and crystallization kinetics.

TEM analysis confirmed the presence of a lamellar morphology whose thickness is similar to that reported for PCL (8 nm). Finally, single crystals of this polymer were prepared, and they displayed flat hexagonal shapes. Studies for determining the exact crystalline structure and unit cell parameters of PHL are in progress.

Polyheptalactones are very promising materials to increase the palette of commercially available polyesters with a potential biodegradable character, given the similarities found between their structure and properties and the various aliphatic polyesters already in use, such as PCL.

## Supporting Information Available

^1^H NMR spectroscopy in CDCl_3_ of η-heptalactone monomer; kinetic plot for the polymerization of η-heptalactone; overlaid RI and UV SEC chromatograms of PHL samples; thermal parameters obtained from non-isothermal experiments; crystallization temperatures as a function of molecular weight of PHL samples; enlargement of the DSC second heating scan; isothermal kinetics data parameters obtained by PLOM and DSC; Avrami fitting parameters; Hoffman–Weeks plots; and histograms of lamellar thickness (PDF)
