# Supplementary material for: Synthesis, Morphology, and Crystallization Kinetics of Polyheptalactone (PHL)
Source: Biomacromolecules. 2023 Jun 21;24(7):3256–67. doi: 10.1021/acs.biomac.3c00305 (PMC10336849; doi:10.1021/acs.biomac.3c00305)
Supplement: Supplementary file 1 — bm3c00305_si_001.pdf [file bm3c00305_si_001.pdf]

Supporting Information

Synthesis, morphology and crystallization kinetics of Polyheptalactone (PHL)

Maria Rosaria Caputo[a], Asier Olmos[a], Bo Li[b], Jorge L. Olmedo-Martínez[a], Anna Malafronte[c], Claudio De Rosa[c], Haritz Sardon[a], Rachel O' Reilly[b], Andrew Dove[b], Alejandro J. Müller[a,d]*

[a] POLYMAT and Department of Polymers and Advanced Materials: Physics, Chemistry and Technology, Faculty of Chemistry, University of the Basque Country UPV/EHU, Paseo Manuel de Lardizábal, 3, 20018 Donostia-San Sebastián, Spain.

[b] University of Birmingham, Edgbaston, Birmingham, B15 2TT, United Kingdom.

[c] Dipartimento di Scienze Chimiche, Università di Napoli Federico II, Complesso Monte S. Angelo, Via Cintia, 80126, Napoli, Italy.

[d] IKERBASQUE, Basque Foundation for Science, Plaza Euskadi 5, 48009 Bilbao, Spain

*Corresponding Author(s): alejandrojesus.muller@ehu.es

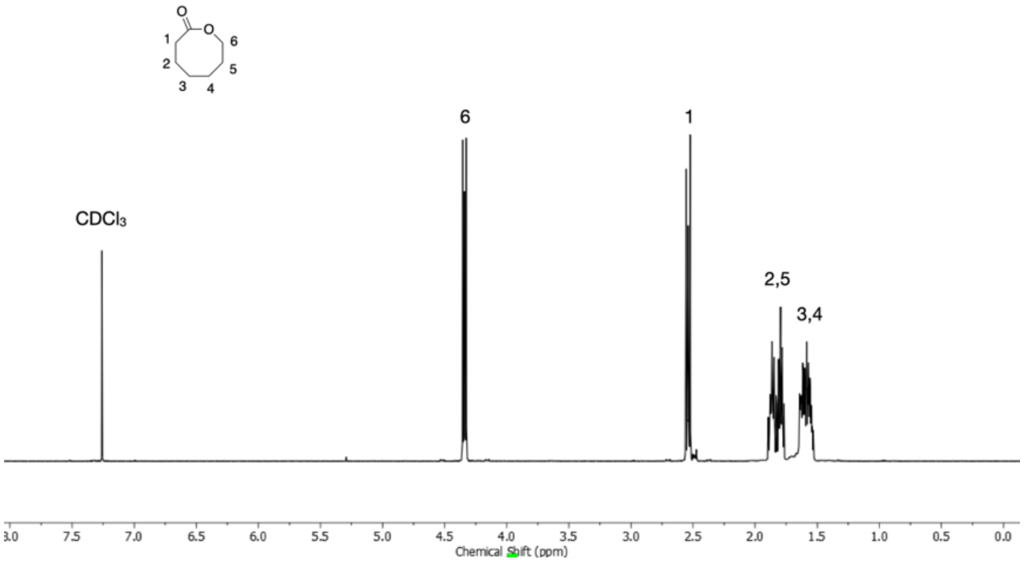

Figure S1. ^{1}H NMR spectroscopy in $CDCl_3$ of η-heptalactone monomer.

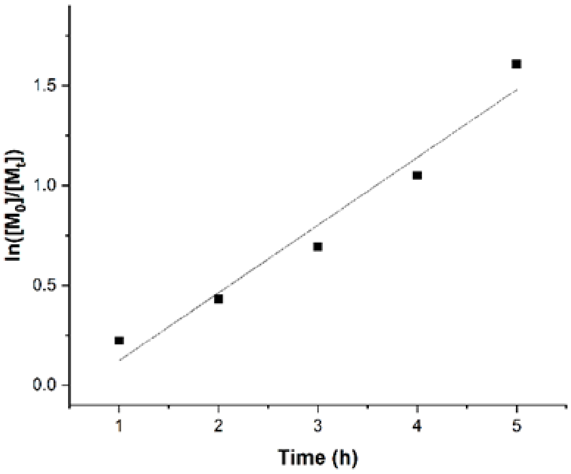

Figure S2. Kinetic plot for the polymerization of η-heptalactone using DPP as a catalyst at room temperature in toluene with $[HL]_0$:$[CTA]_0$:$[cat.]_0$ = 100:1:1 and initial monomer concentration = 4 M.

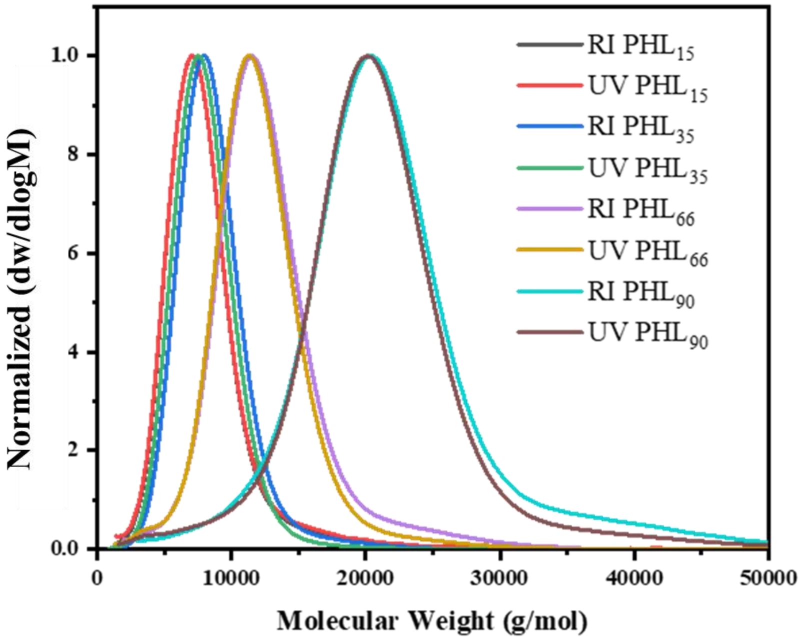

Figure S3. Overlaid RI and UV (λ = 309 nm) SEC chromatograms of PHL using $CHCl_3$ with 0.1% TFE as an eluent with polystyrene (PS) standards.

Table S1. Thermal parameters obtained from non-isothermal crystallization

	T_g (°C)	T_c (°C)	T_m (°C)	ΔH_c (J/g)	ΔH_m (J/g)	X_c (%)
**PHL 15**	-39.3	36.1	55.1	86	84	43
**PHL 35**	-42.5	38.0	58.8	77	79	41
**PHL 66**	-41.1	41.9	61.3	91	90	46
**PHL 90**	-39.7	42.3	61.7	91	93	48

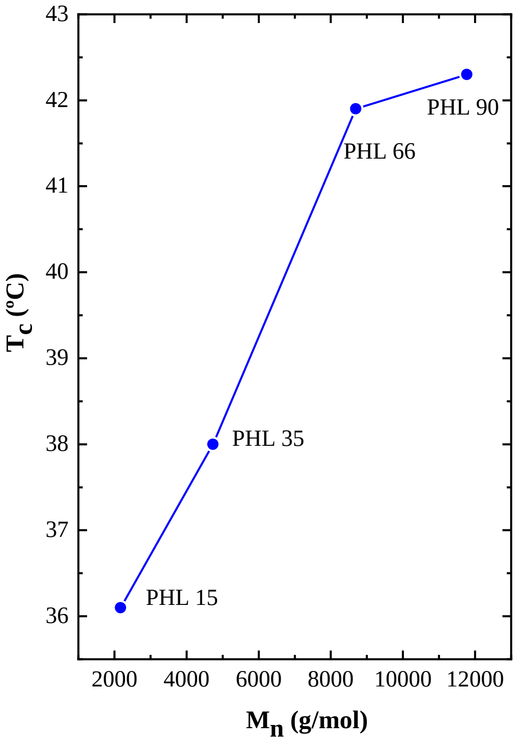

Figure S4. Crystallization temperatures as a function of molecular weight of PHL samples; the solid blue line is an arbitrary fit.

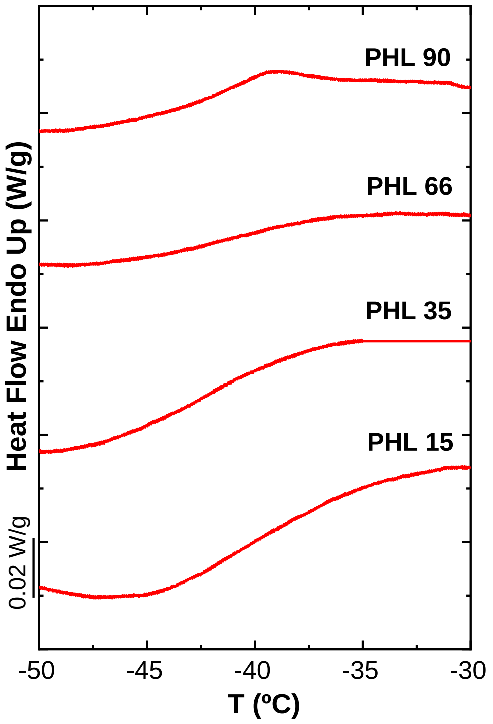

Figure S5. Glass transition for the four PHL samples.

Table S2. Isothermal kinetics data parameters described in the Paragraph 3.4 derived from experimental results obtained by PLOM.

Sample	G^0(cm/s)	K^G_g (K^2)	σ (erg/cm^2)	σ_e (erg/cm^2)	q (erg)	R^2
PHL 15	753	3.55x10^5	11.16	136.19	5.03x10^{-13}	0.987
PHL 35	808	1.18x10^5	11.16	133.44	4.92x10^{-13}	0.946
PHL 66	854	6.12x10^4	11.16	69.74	2.57x10^{-13}	0.939
PHL 90	902	2.92x10^4	11.16	33.27	1.34x10^{-13}	0.932

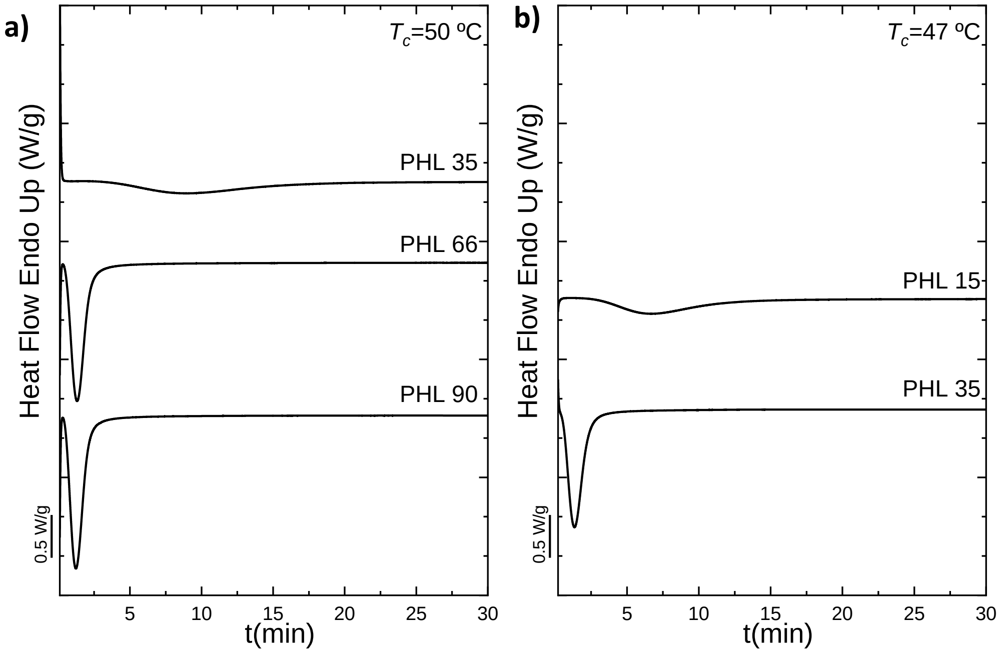

Figure S6. DSC scans recorded during isotherms at (a) T_c=50 ºC for PHL 35, PHL 66, PHL 90 and at (b) T_c= 47 ºC for PHL 15 and PHL 35.

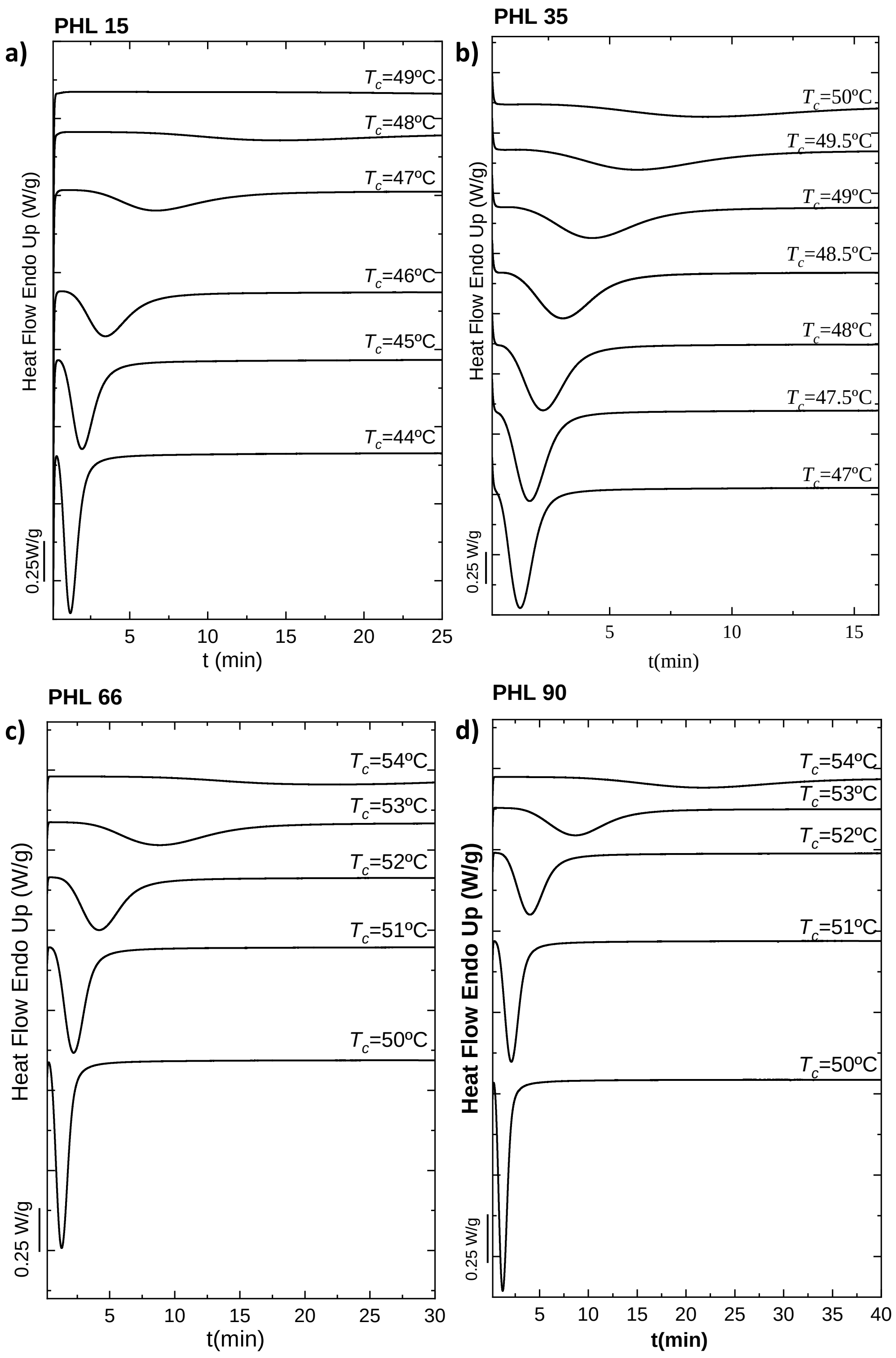

Figure S7. DSC scans recorded during isotherms for PHL 15 (a) PHL 35 (b), PHL 66 (c) and PHL 90 (d).

Table S3. Isothermal kinetics data parameters described in the Paragraph 3.5 derived from experimental results obtained by DSC.

Sample	$1/\tau_0$(1/s)	K^{τ}_g (K^2)	σ (erg/cm^2)	σ_e (erg/cm^2)	q (erg)	R^2
PHL 15	1.22x10^9	5.53x10^5	11.16	193.19	7.12x10^{-13}	0.987
PHL 35	9.98x10^9	3.71x10^5	11.16	173.03	6.39x10^{-13}	0.993
PHL 66	6.26x10^6	9.02x10^4	11.16	115.7	4.27x10^{-13}	0.99
PHL 90	5.33x10^6	4.61x10^4	11.16	109.43	4.04x10^{-13}	0.99

Table S4. Avrami fitting parameters obtained by the free App [1] for PHL 15.

T_c (°C)	t_0 (min)	n	K (min^{-n})	$K^{1/n}$ (min^{-1})	R	$\tau_{50\%exp}$(min)	$\tau_{50\%Theo}$ (min)
44	0.352	2.85	8.00 x 10^{-01}	5.30 x 10^{-01}	1.000	0.951	0.978
45	0.440	3.14	1.37 x 10^{-01}	1.94 x 10^{-03}	1.000	1.674	1.742
46	0.573	3.50	1.26 x 10^{-02}	2.25 x 10^{-07}	0.999	3.140	3.327
47	1.638	3.04	3.80 x 10^{-03}	4.39 x 10^{-08}	1.000	5.535	5.730
48	3.272	3.44	1.58 x 10^{-04}	8.38 x 10^{-14}	0.999	11.425	11.933
49	19.102	2.17	1,23 x 10^{-03}	4.84 x 10^{-07}	1.000	18.504	18.646

Table S5. Avrami fitting parameters obtained by the free App [1] for PHL 35.

T_c (°C)	t_0 (min)	n	K (min^{-n})	$K^{1/n}$ (min^{-1})	R	$\tau_{50\%exp}$(min)	$\tau_{50\%Theo}$ (min)
47	0.350	2.85	5.59 x 10^{-01}	1.91 x 10^{-01}	1.000	1.079	1.092
47.5	0.418	2.95	2.47 x 10^{-01}	1.61 x 10^{-02}	1.000	1.418	1.442
48	0.705	2.6	1.63 x 10^{-01}	8.95 x 10^{-03}	0.999	1.745	1.743
48.5	1.007	2.52	8.24 x 10^{-02}	1.85 x 10^{-03}	0.999	2.327	2.316

49	1.022	2.85	1.80×10^{-02}	1.07×10^{-05}	1.000	3.599	3.656
49.5	1.848	2.58	1.23×10^{-02}	1.18×10^{-05}	1.000	4.751	4.772
50	2.167	2.83	2.39×10^{-03}	3.81×10^{-08}	1.000	7.394	7.563

Table S6. Avrami fitting parameters obtained by the free App [1] for PHL 66.

T_c (°C)	t_0 (min)	n	K (min^{-n})	$K^{1/n}$ (min^{-1})	R	$\tau_{50\%exp}$(min)	$\tau_{50\%Theo}$ (min)
50	0.353	2.86	6.37×10^{-01}	2.75×10^{-01}	1.000	1.036	1.054
51	0.413	3.31	7.56×10^{-02}	1.94×10^{-04}	1.000	1.955	2.024
52	0.997	3.03	1.59×10^{-02}	3.55×10^{-06}	1.000	3.471	3.555
53	2.237	2.96	1.97×10^{-03}	$9,81 \times 10^{-09}$	1.000	7.242	7.493
54	5.693	2.91	1.81×10^{-04}	1.29×10^{-11}	1.000	17.042	17.995

Table S7. Avrami fitting parameters obtained by the free App [1] for PHL 90.

T_c (°C)	t_0 (min)	n	K (min^{-n})	$K^{1/n}$ (min^{-1})	R	$\tau_{50\%exp}$(min)	$\tau_{50\%Theo}$ (min)
50	0.353	2.75	7.94×10^{-01}	5.30×10^{-01}	1.000	0.952	0.969
51	0.413	3.23	1.02×10^{-01}	6.28×10^{-04}	1.000	1.810	1.640
52	0.705	3.37	9.98×10^{-03}	1.70×10^{-07}	1.000	3.519	3.627
53	1.647	3.37	7.70×10^{-04}	3.22×10^{-11}	1.000	7.507	7.736
54	5.985	3.01	1.40×10^{-04}	2.51×10^{-12}	0.999	16.901	16.922

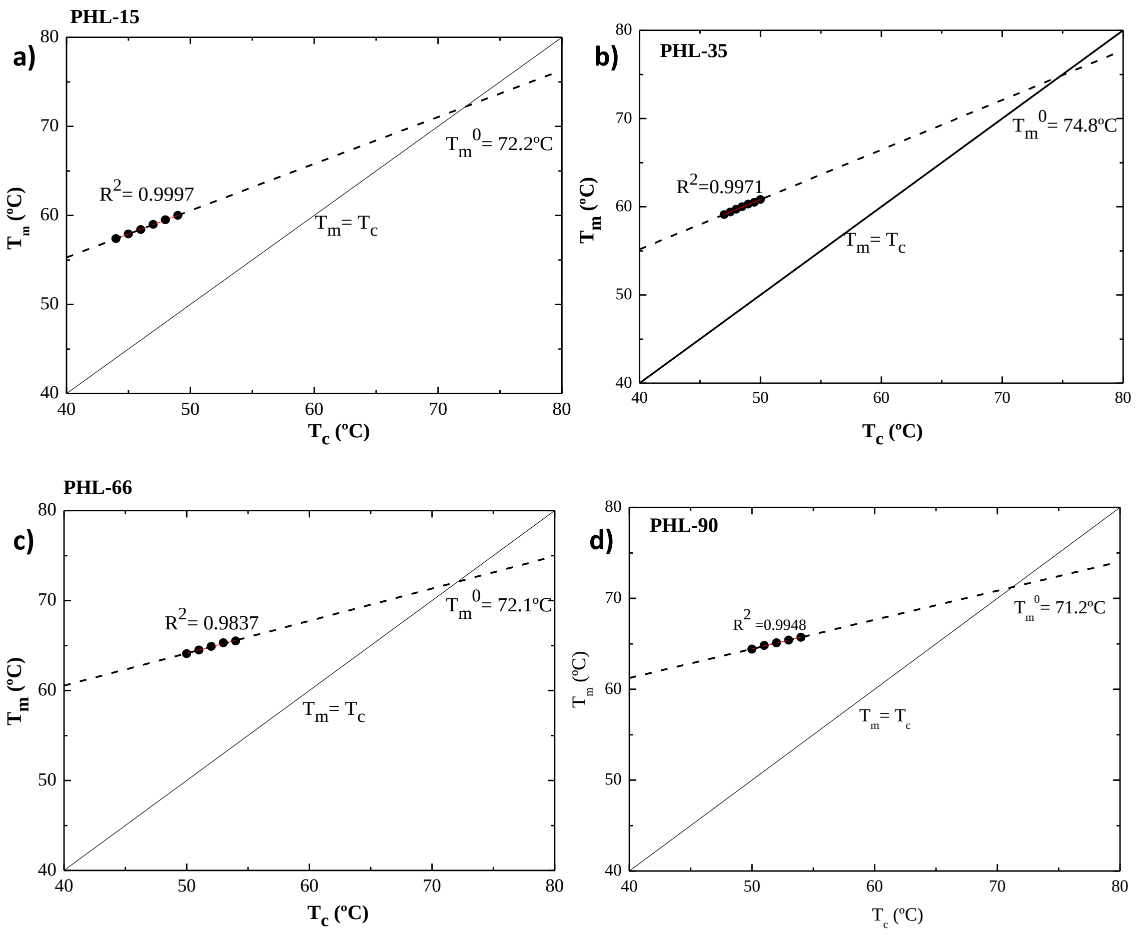

Figure S7. Hoffman-Weeks plots for PHL 15 (a), PHL 35 (b), PHL 66 (c), and PHL 90K (d).

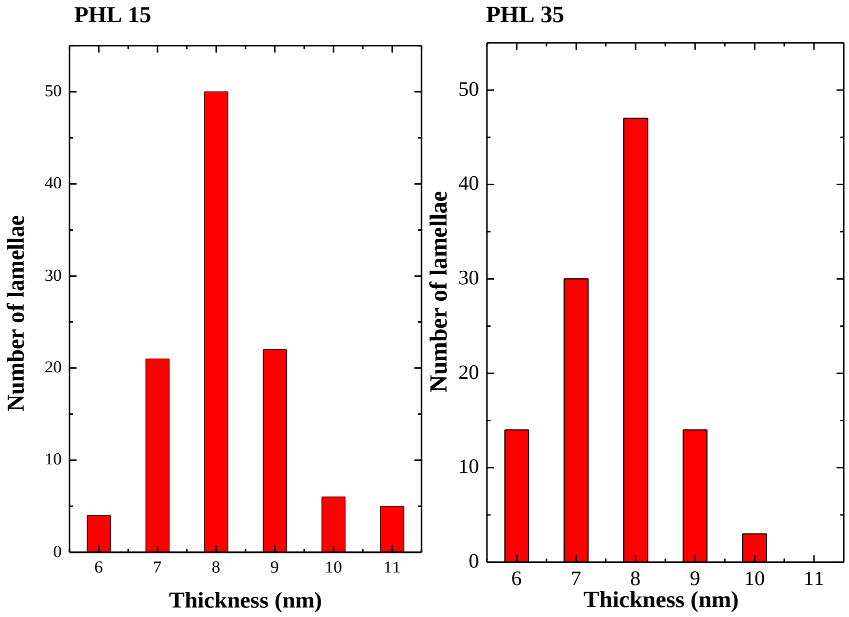

Figure S8. Distribution of the measured lamellae thickness of PHL 15 and PHL 35.

(1) Pérez-Camargo, R. A.; Liu, G.; Wang, D.; Müller, A. J. Experimental and Data Fitting Guidelines for the Determination of Polymer Crystallization Kinetics. *Chinese J. Polym. Sci* **2022**, *40*, 1–34. https://doi.org/https://doi.org/10.1007/s10118-022-2724-2.
